# Pro‐197 mutations confer resistance to thiencarbazone but not to foramsulfuron in ALS‐resistant *Poa annua* populations

**DOI:** 10.1002/ps.71051

**Published:** 2026-06-21

**Authors:** Vijaya Bhaskar Alwarnaidu Vijayarajan, Iniya Vijayakumar, Rafa Ruiz‐Partida, Fabian Runge, Joel Torra, Jorge Lozano‐Juste, Patrick Dermot Forristal

**Affiliations:** ^1^ Crop Science Department Teagasc Oak Park Research Centre Carlow Ireland; ^2^ Department of Applied Sciences South East Technological University, Wexford Campus Wexford Ireland; ^3^ Instituto de Biología Molecular y Celular de Plantas (IBMCP) Universitat Politècnica de València (UPV), Consejo Superior de Investigaciones Científicas (CSIC) Valencia Spain; ^4^ IDENTXX GmbH Stuttgart Germany; ^5^ Department of Agricultural and Forest Sciences and Engineering University of Lleida – Agrotecnio CERCA Centre Lleida Spain

**Keywords:** annual bluegrass, co‐formulation, docking, foramsulfuron, Pro‐197, structural modelling, target‐site mutations, thiencarbazone, Trp‐574

## Abstract

**BACKGROUND:**

*Poa annua* is currently a lower‐priority arable weed in Ireland; however, increasing target‐site resistance to acetolactate synthase (ALS) inhibitors and declining availability of non‐ALS herbicides highlight emerging control challenges. ALS‐inhibiting co‐formulations of foramsulfuron + thiencarbazone have been specifically developed for use in herbicide‐tolerant beet varieties. While foramsulfuron can control grass weeds carrying specific target‐site mutations, its efficacy within the co‐formulation on ALS‐resistant populations remains unknown. We addressed this knowledge gap using genetic characterisation, dose–response and interaction analyses, and ligand docking modelling in 12 independently evolved, ALS‐resistant, *Poa annua* populations carrying Pro‐197 or Trp‐574 mutations.

**RESULTS:**

All Pro‐197 variants (Thr, Gln or Ala) were thiencarbazone‐resistant (resistance index (RI) = 20.9–33.3) but remained foramsulfuron‐sensitive (2.5–4), while the co‐formulation showed reduced efficacy (5.5–6.2). Interaction analyses showed no overall deviation from additivity for Pro‐197 populations (*P* > 0.05), although antagonism occurred at reduced rates (*P* < 0.05). Structural modelling and ligand docking analyses indicated that most Pro‐197 substitutions did not alter the ALS catalytic tunnel. Foramsulfuron retained extensive hydrogen‐bonding and electrostatic interactions, with a more favourable binding free energy than thiencarbazone, which showed fewer interactions and a less favourable free energy. Trp‐574‐Leu variants exhibited cross‐resistance to all treatments, consistent with disruption of key binding interactions.

**CONCLUSION:**

This study demonstrates that foramsulfuron + thiencarbazone efficacy on Pro‐197‐resistant *Poa annua* populations is poor, despite these populations being sensitive to foramsulfuron used alone. Consequently, this co‐formulation, developed for ALS‐tolerant beet, should be restricted to fields without ALS‐resistant weeds to ensure effective in‐crop control and avoid selection for resistance. © 2026 The Author(s). *Pest Management Science* published by John Wiley & Sons Ltd on behalf of Society of Chemical Industry.

## Graphical abstract

Foramsulfuron remains effective on Pro‐197 *Poa annua*, but co‐formulation with thiencarbazone reduces efficacy due to limited activity and antagonism. Therefore, foramsulfuron + thiencarbazone should be used in fields without ALS‐resistant weeds to ensure effective in‐crop control.

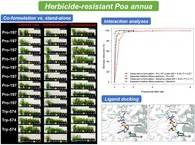

## 1 INTRODUCTION

In Ireland, *Poa annua* L. (annual bluegrass or annual meadow grass), the most widely distributed grass weed, is normally considered a low‐priority weed in arable crops.^1^ It is primarily a self‐pollinating allotetraploid (2*n* = 28), with high reproductive capacity and seed viability, forming a persistent seed bank.^2^, ^3^ A naturally inherited Ile‐1781 mutation confers resistance to most acetyl‐CoA carboxylase (ACCase) inhibitors, except clethodim (used in winter oilseed rape or beet).^4^ Over the past decade, reliance on acetolactate synthase (ALS) inhibitors for post‐emergence grass weed control in winter wheat, more frequently targeting *Bromus sterilis* L. (sterile brome) primarily with *Poa annua* control secondary, has selected cross‐resistance in *Poa annua* to sulfonylureas (SUs; mesosulfuron, iodosulfuron), sulfonylamino‐carbonyl‐triazolinones (SCTs; propoxycarbazone, thiencarbazone) and triazolopyrimidines (TPs; pyroxsulam).^5^ Wet autumns (with monthly rainfall ranging from 78.6 to 126.2 mm) and fewer pre‐emergence spray opportunities, along with the loss of autumn post‐emergence isoproturon (photosynthesis inhibitor) in the European Union (EU) in 2017, have contributed to this over‐reliance.^4^, ^5^ Most cases of resistance in *Poa annua* involve target‐site resistance (TSR) mutations at Pro‐197 or Trp‐574 on the ALS enzyme.^4^, ^5^ Trp‐574 mutations confer broad ALS cross‐resistance, whereas Pro‐197 substitutions usually confer SU resistance, with variable effects depending on the amino acid change, as seen in our recent study.^5^

Understanding the biochemical basis of ALS inhibition is essential for interpreting resistance mechanisms and herbicide efficacy.^6^ ALS is an enzyme that catalyses the first step in the biosynthesis of the branched‐chain amino acids leucine, isoleucine and valine in plants and microorganisms.^7^, ^8^ The plant holoenzyme is composed of regulatory and catalytic subunits, in an α_4_β_4_ configuration. The catalytic subunit, which contains the cofactors FAD (flavin adenine dinucleotide), ThDP (thiamine diphosphate) and magnesium ion (Mg^2+^), is the primary target of herbicides.^9^ Catalytic site formation occurs upon protein dimerisation, which simultaneously generates a tunnel that enables substrate access to the active site. ALS‐inhibiting herbicides act by binding within this tunnel, thereby blocking substrate access.^7^

In Ireland, broad‐leaved crops such as oilseed rape (11 500 ha), beans (8200 ha) and fodder beet (9800 ha) are often grown in rotation with cereals, typically 1 year in every 5 years.^10^, ^11^ These crop types enable the control of grass weeds and cereal volunteers through the use of a wide range of graminicide herbicides.^1^ However, the effectiveness of this option has recently been severely limited by the occurrence of herbicide‐resistant populations of wild oats (*Avena fatua* L.),^12^ blackgrass (*Alopecurus myosuroides* L.)^1^, ^11^ and Italian ryegrass (*Lolium multiflorum* L.),^1^, ^11^ along with naturally resistant *Poa annua*.^4^, ^5^ Within the same time period, weed control in beet crops has been made more challenging by the loss of the photosynthesis inhibitor desmedipham in 2019, and the broadleaf‐active ALS SU herbicide triflusulfuron in 2024 in the EU.^13^

The use of herbicides containing active ingredients that would normally be unsafe for the target crop, along with crop varieties specifically bred to incorporate tolerance to these active ingredients, has been developed for a number of crops to give more herbicide options.^14^, ^15^ A recent development is ALS‐tolerant beet, which allows the use of ALS‐inhibiting (SU + SCT) foramsulfuron + thiencarbazone co‐formulated herbicides, and has been approved for use in Ireland since 2021.^16^, ^17^ The standard use rate in tolerant beet crops is 50 g foramsulfuron ha^−1^ + 30 g thiencarbazone ha^−1^ (a higher SCT dose than permitted in cereal crops, where the rate is 7.5 g ha^−1^) and primarily targets susceptible broadleaf weeds but also offers activity on some susceptible grasses, including *Poa annua* and *Echinochloa crus‐galli* L. (barnyard grass).^13^, ^17^ Although this co‐formulation for tolerant‐beet varieties is not specifically intended for grass weed control in this region, previous research has shown contrasting responses of ALS‐resistant grass weeds to its component herbicides, reflecting the mutation‐ and chemistry‐specific nature of ALS resistance.^18^

Unlike many SUs and other ALS herbicide chemistries that lose activity against Pro‐197 variants,^19^ foramsulfuron has shown potential to control Pro‐197‐resistant grass weeds. In our recent study, a *L. multiflorum* population harbouring Pro‐197 mutations remained susceptible to foramsulfuron despite showing resistance to most other SU herbicides (Vijayakumar *et al*. unpublished; Supporting Information, Fig. S1). Similar differential responses to foramsulfuron and the SU herbicide rimsulfuron have been observed in a Pro‐197 mutant *Poa annua* population.^20^ However, the mechanism underlying this response is not fully understood. While Pro‐197 mutants remain susceptible, foramsulfuron resistance has been found in *Poa annua* and over 15 other species, predominately associated with Trp‐574 mutations.^19^, ^20^, ^21^, ^22^ In addition, our work also showed that Pro‐197 mutant *Poa annua* populations were resistant to thiencarbazone applied alone or in co‐formulation with mesosulfuron at standard cereal use rates (Fig. S2).^5^ These contrasting sensitivities to SU and SCT chemistries raise concerns regarding the performance of a foramsulfuron + thiencarbazone co‐formulation, especially in fields where ALS‐resistant grass weeds are present.

Given the increasing prevalence of Pro‐197‐associated resistance in *Poa annua* in Ireland, the risk of deregistration of effective residual actives, limited cultural and non‐chemical control options, and the absence of a stand‐alone foramsulfuron herbicide in the region, evaluating this co‐formulation is crucial. It may add an alternative in‐crop option along with the ACCase inhibitor clethodim, for managing *Poa annua* populations that would have been considered ALS‐resistant in beet crops. The combined activity of foramsulfuron + thiencarbazone relative to its individual active ingredients as well as the impact of different Pro‐197 mutations on herbicide binding remain unexplored. Using independently evolved ALS‐resistant populations, this study aimed to: (a) assess the response of *Poa annua* carrying Pro‐197 or Trp‐574 mutations to the co‐formulated foramsulfuron + thiencarbazone, (b) compare the efficacy of the co‐formulation with each active ingredient applied individually, and (c) explore the herbicide binding interactions and the effects of Pro‐197 substitutions using ligand docking simulations.

## 2 MATERIAL AND METHODS

### 2.1 Plant materials

Twelve resistant *Poa annua* (POAAN‐R) populations collected in 2023 and 2024 were used in this study (Table 1). The source fields for these populations had no history of foramsulfuron + thiencarbazone application but had been subjected to intensive use of mesosulfuron + iodosulfuron in winter wheat. Initial screening at the standard application rate of mesosulfuron + iodosulfuron (data not shown) and subsequent dose–response experiments on ten POAAN‐R populations with sufficient seed quantity, confirmed evolved ALS resistance (Fig. S3). A previously confirmed ALS‐sensitive *Poa annua* population (WeberSeeds Botany and Ethnobotany, Vaals, The Netherlands) was used as the herbicide‐sensitive (S) reference population.^4^, ^5^

**Table 1:** Population origin of *Poa annua* (POAAN‐R) used in dose–response analysis with acetolactate synthase (ALS)‐inhibiting sulfonylurea (SU) and sulfonylamino‐carbonyl‐triazolinone (SCT) herbicides (foramsulfuron and thiencarbazone), applied as stand‐alone and co‐formulated products

Population code	Field position	County	Country	Harvested crop	Year
POAAN‐R1	52°26′ N–6°51′ W	Wexford	Ireland	Winter wheat	2023
POAAN‐R1.1	52°33′ N–6°31′ W	Wexford	Ireland	Winter wheat	2023
POAAN‐R1.2	53°35′ N–6°26′ W	Dublin	Ireland	Winter wheat	2024
POAAN‐R1.3	53°87′ N–6°74′ W	Meath	Ireland	Winter wheat	2024
POAAN‐R2	54°37′ N–6°35′ W	Armagh	Northern Ireland, UK	Winter wheat	2023
POAAN‐R3	53°19′ N–6°98′ W	Kildare	Ireland	Winter wheat	2023
POAAN‐R3.1	53°95′ N–6°55′ W	Louth	Ireland	Winter wheat	2024
POAAN‐R4	53°95′ N–6°55′ W	Louth	Ireland	Winter wheat	2024
POAAN‐R5	51°90′ N–8°45′ W	Cork	Ireland	Winter wheat	2024
POAAN‐R5.1	53°64′ N–6°88′ W	Meath	Ireland	Winter wheat	2024
POAAN‐R5.2	53°40′ N–6°27′ W	Dublin	Ireland	Winter wheat	2024
POAAN‐R6	52°27′ N–6°44′ W	Wexford	Ireland	Winter wheat	2023

### 2.2 Genetic analyses

Leaf samples from R (*n* = 8, treated survivors) and S (*n* = 8, untreated controls) populations were collected from the initial screening and sent to IDENTXX GmbH (Stuttgart, Germany) for DNA isolation, amplification, and sequencing. DNA extraction and subsequent polymerase chain reactions (PCRs) were done as described before in previous work.^5^ Briefly, gene fragments encompassing the two known mutation sites Pro‐197 and Trp‐574 of the *ALS* gene, and the inherited Ile‐1781 mutation of the *ACCase* gene were amplified and single nucleotide polymorphisms (SNPs) were detected using pyrosequencing.

*Poa annua* contains two ALS isoforms, *ALS1* and *ALS2* (GenBank: KT346395.1, KT346396.1), which are thought to be equally expressed and contribute to ALS activity^23^; both are capable of carrying resistance mutations. Mutations detected by pyrosequencing may therefore reflect presence in one or both isoforms in either heterozygous or homozygous form. To identify mutation locations, isoform‐specific PCRs were carried out. Only positions that showed a mutation in the pyrosequencing were analysed (see Section 3.1). PCR followed the protocol previously described by Alwarnaidu Vijayarajan *et al*.^5^ using the primer sets listed therein, and the amplicons were sent to Seqlab (Göttingen, Germany) for Sanger sequencing. Resulting sequences were analysed using Geneious v9.1.8.

### 2.3 Dose–response to ALS SU and SCT inhibitors

The dose–response experiments were carried out on R and S seedlings during the winters of 2023/2024 (populations POAAN‐R1 to POAAN‐R5) and 2024/2025 (populations POAAN‐R1.1, POAAN‐R1.2, POAAN‐R1.3, POAAN‐R3.1, POAAN‐R5.1, POAAN‐R5.2 and POAAN‐R6).

Seeds were sown in 90 mm × 90 mm × 100 mm pots filled with a standard Progrow (Enrich Environmental Ltd, County Meath, Ireland) soil mix containing 58% sand, 22% silt and 20% clay and supplemented with 2 g L^−1^ of Osmocote Mini (National Agrochemical Distributors Ltd, Blake's Cross Lusk, Ireland). At the two‐to‐four leaf stage (BBCH 12–14), plants were treated with a range of doses of three ALS inhibitors (Table 2). Each replicate pot contained six to eight plants. Herbicides were applied using a Generation III Research Track Sprayer (DeVries Manufacturing, Hollandale, MN, USA) with a Teejet 8002‐EVS flat fan nozzle, delivering an output of 200 L ha^−1^ at a pressure of 250 kPa and speed of 1.2 m s^−1^. For stand‐alone foramsulfuron applications (Table 2), a spray volume of 250 L ha^−1^ was used according to label recommendations. Treated foliage was allowed to dry before pots were returned to the glasshouse, and watering resumed after 24 h.

**Table 2:** Acetolactate synthase (ALS)‐inhibiting sulfonlyurea (SU) and sulfonylamino‐carbonyl‐triazolinone (SCT) herbicides, applied as co‐formulated or stand‐alone, used for dose–response assays in resistant (R) and sensitive (S) populations of *Poa annua*

Type	Chemical families	Trade name^†^	Source	Active ingredient (ai)	Dose (g a.i. ha^−1^)
Co‐formulated	SU + SCT	Conviso® One	Bayer CropScience Ltd, Cambridge, UK	Foramsulfuron + thiencarbazone	3.1 + 1.9, 6.3 + 3.8, 12.5 + 7.5, 25 + 15, **50** + **30**, 75 + 45, 100 + 60, 200 + 120, 400 + 240 and 0
Stand‐alone	SU	Cubix®	Bayer CropScience Ltd, Barcelona, Spain	Foramsulfuron	2.8, 5.6, 11.3, 22.5, **45**, 67.5, 90, 180, 360 and 0
	SCT	TCM	Bayer CropScience Ltd, Cambridge, UK	Thiencarbazone	1.9, 3.8, 7.5, 15, **30**, 45, 60, 120, 240 and 0

^†^ Cubix is not registered for use in Ireland. TCM (Spec. no. SP102000013889; batch 2100034201) was provided by Bayer CropScience Ltd, UK, strictly for research purposes; no commercial product exists under this name. Conviso One and Cubix are formulated as oil dispersion (OD), whereas TCM is formulated as wettable granules (WG).

*Note:* The recommended label rates for use are highlighted in bold.

For the R populations, dose ranges of 0.25× to 8× the recommended label rate were used, while for the S population, the range was 0.0625× to 2× the recommended rate. Due to limited seed quantity, four replicates per dose were used for the co‐formulated experiment, and three for the stand‐alone experiments. At 30 days post‐treatment, aboveground plant material was harvested from each replicate and shoot fresh weight recorded. Shoot biomass for each replicate was expressed as the percentage of the mean fresh biomass of the corresponding untreated controls. All plants were grown in a glasshouse with a 16 h photoperiod, supplemented with artificial lighting to maintain a minimum light intensity of 250 μmol m^−2^ s^−1^, and a temperature regime of 18 °C/12 °C (day/night).

### 2.4 ALS enzyme modelling and ligand docking

The *Poa annua* ALS enzyme (PoaALS) amino acid sequence was obtained from the National Centre for Biotechnology Information (NCBI, ALE27653.1). Homology models of PoaALS and its variants were generated using the SWISS‐MODEL server,^24^ with the crystal structure of ALS from *Arabidopsis thaliana* (PDB ID: 3E9Y) as a template. Model quality metrics and Ramachandran plots were generated using the Swiss model structure assessment web server (Supporting Information, Fig. S4 and Table S1).^25^ Model visualisation and sequence‐structure three‐dimensional (3D) alignment were performed using Chimera.^26^

Ligand interaction diagrams of PoaALS in complex with thiencarbazone and foramsulfuron were generated from *in silico* docking experiments using Maestro (Schrödinger Release 2024‐4). The chemical structures of the ligands were prepared with the LigPrep method (Schrödinger Release 2024‐4) with Epic classic ionisation states in the pH range of 7.4 ± 0.2, generation of tautomers and retention of specified chirality, using the OPLS4 force field for optimisation. ALS dimers from the structural models were minimised and prepared for docking using Protein Preparation Wizard in Maestro (Schrödinger Release 2024‐4) with default settings (minimisation to a maximum root mean square deviation (RMSD) of 0.3 Å, OPLS4 forcefield, deletion of waters within 5 Å of heteroatoms, water orientation sampling and PROPKA‐based protonation state optimisation). The coordinates of monosulfuron bound to ALS models were used as the centroid for receptor grid generation and Glide standard precision was used for docking simulations (Schrödinger Release 2024‐4). Molecular Mechanics/Generalised Born Surface Area (MM/GBSA) calculations to estimate binding energies were performed using Prime, Maestro (Schrödinger Release 2024‐4) with default setting (OPLS4 force field and the VSGB (Volume Surface Generalised Born) implicit solvation model). Tunnel characterisation was performed with MOLE 2.0,^27^ using the co‐crystallised ThDP as the starting point and default parameters (bottleneck radius = 1.25, origin radius = 5.00, surface cover radius = 10.0, bottleneck length = 0.00, cut‐off radius = 0.90 and minimum length for tunnel and pore = 0.00). Finally, comparisons of catalytic tunnels among PoaALS variants were conducted using ChimeraX.

### 2.5 Statistical analysis

Data were analysed using R (version 3.6.3). Dose–response models were fitted to shoot fresh weight data using the *DRC* package.^28^ A three‐parameter log‐logistic model was selected through the lack‐of‐fit *F‐*tests (*P* > 0.05) to model the data of co‐formulated foramsulfuron + thiencarbazone and stand‐alone foramsulfuron and thiencarbazone treatments. The fitted models estimated the growth rate GR_50_ (i.e., the effective dose rate required to reduce growth by 50% relative to untreated plants). Following a lack of significant differences (*P* > 0.05, with some exceptions) between estimated GR_50_ values using *t*‐statistics from the *compParm*() function, populations were grouped by mutation type (Pro‐197 or Trp‐574); and the data were modelled using the same three‐parameter log‐logistic model. Resistance index (RI) values were calculated as the ratio of the GR_50_ of R populations to that of the sensitive reference.

To assess potential interactions between foramsulfuron and thiencarbazone, mixture effects were evaluated using the Additive Dose Model (ADM).^29^ Dose–response curves fitted with the three‐parameter log‐logistic model were compared to additive effects predicted by the Bliss Independence model,^30^, ^31^ which assumes independent herbicide action. The expected biomass reduction at dose *d* was calculated as: *R*_Bliss_(*d*) = *R*_foram_(*d*) + *R*_thien_(*d*) − ((*R*_foram_(*d*) × *R*_thien_(*d*))/100), where *R* values represent biomass reduction (%), calculated as 100 minus fresh weight (% of untreated control) for each stand‐alone herbicide. Interaction effects at dose *d*, Δ(*d*) = *O*_mix_(*d*) − *R*_Bliss_(*d*), were derived by comparing the observed biomass reduction from the co‐formulation (*O*_mix_) to the predicted values. Interaction was classified as synergistic when the co‐formulation caused greater biomass reduction than predicted, antagonistic when it caused less, and additive when the effects matched the Bliss prediction. A paired *t*‐test was used to assess the significance of differences between observed and expected values across dose rates.

## 3 RESULTS

### 3.1 Genetic analyses

Pyrosequencing and sequence alignment analyses confirmed that all 12 POAAN‐R populations had mutations at positions Pro‐197 and/or Trp‐574 in the ALS gene, with mutations arising independently in either the *ALS1* or *ALS2* homologues (Table 3). No mutations at these positions were detected in the S population. Nine POAAN‐R populations predominantly harboured Pro‐197 substitutions, while the remaining three carried Trp‐574 mutations (Table 3). Pyrosequencing and sequence alignment data for POAAN‐R1, POAAN‐R2, POAAN‐R3, POAAN‐R4 and POAAN‐R5 have been reported previously.^5^

**Table 3:** Amino acid (aa) substitutions detected at acetolactate synthase (ALS) Pro‐197 and Trp‐574 positions in *Poa annua* populations

	ALS pyrosequencing results				*ALS1*				*ALS2*			
	Pro (CCG)‐197		Trp (TGG)‐574		Pro (CCG)‐197		Trp (TGG)‐574		Pro (CCG)‐197		Trp (TGG)‐574	
Population^†^	Codon	aa	Codon	aa	Codon	aa	Codon	aa	Codon	aa	Codon	aa
POAAN‐R1	C/A‐CG (8)	**Pro/Thr**	TGG (8)	Trp	CCG (8)	Pro			ACG (8)	**Thr**		
POAAN‐R1.1	C/A‐CG (8)	**Pro/Thr**	TGG (8)	Trp	CCG (8)	Pro			ACG (8)	**Thr**		
POAAN‐R1.2	C/A‐CG (8)	**Pro/Thr**	TGG (8)	Trp	ACG (8)	**Thr**			CCG (8)	Pro		
POAAN‐R1.3	C/A‐CG (8)	**Pro/Thr**	TGG (8)	Trp	ACG (8)	**Thr**			CCG (8)	Pro		
POAAN‐R2	C‐C/A‐G (8)	**Pro/Gln**	TGG (8)	Trp	CAG (8)	**Gln**			CCG (8)	Pro		
POAAN‐R3	C‐C/A‐G (5), C/A‐CG (3)	**Pro/Gln, Pro/Thr**	TGG (8)	Trp	CCG (3), CAG (5)	Pro, **Gln**			CCG (5), ACG (3)	Pro, **Thr**		
POAAN‐R3.1	C/A‐CG (2), C‐C/A‐G (5), C/A‐C/A‐G (1)	**Pro/Thr, Pro/Gln, Pro/Gln/Thr**	TGG (8)	Trp	CCG (2), CAG (5), C‐C/A‐G (1)	Pro **Gln Pro/Gln**			CCG (5), ACG (2), C/A‐CG (1)	Pro, **Thr, Pro/Thr**		
POAAN‐R4	C/A‐CG (7)	**Pro/Thr**	T‐G/T‐G (1)	**Trp/Leu**	CCG (8)	Pro	TGG (8)	Trp	ACG (7)	**Thr**	TGG (7), TTG (1)	Trp, **Leu**
POAAN‐R5	CCG (8)	Pro	T‐G/T‐G (8)	**Trp/Leu**			TGG (8)	Trp			TTG (8)	**Leu**
POAAN‐R5.1	CCG (8)	Pro	T‐G/T‐G (8)	**Trp/Leu**			TGG (8)	Trp			T‐G/T‐G (8)	**Trp/Leu**
POAAN‐R5.2	CCG (8)	Pro	T‐G/T‐G (8)	**Trp/Leu**			TTG (8)	**Leu**			TGG (8)	Trp
POAAN‐R6	A/G‐CG (8)	**Thr/Ala**	TGG (8)	Trp	GCG (8)	**Ala**			ACG (8)	**Thr**		
Sensitive	CCG (8)	Pro	TGG (8)	Trp								

^†^ Pyrosequencing and sequence alignment data for POAAN‐R1, POAAN‐R2, POAAN‐R3, POAAN‐R4 and POAAN‐R5 were reported previously.^5^

*Note:* Eight plants were analysed per population. The number of plants carrying specific mutations is indicated in parenthesis. Sequence alignment analysis (*ALS1* and *ALS2*) was conducted only for ALS positions where mutations were identified by pyrosequencing. Mutant aa substitutions are presented in bold font.

The POAAN‐R1 group (POAAN‐R1, POAAN‐R1.1, POAAN‐R1.2 and POAAN‐R1.3) had all plants with homozygous Pro‐197‐Thr substitutions in either the *ALS1* or *ALS2* isoform (Table 3). In POAAN‐R2, all plants had homozygous Pro‐197‐Gln substitutions in *ALS1*. The POAAN‐R3 and POAAN‐R3.1 populations showed a combination of Pro‐197‐Thr and Pro‐197‐Gln substitutions. In one population, five plants carried homozygous Pro‐197‐Gln mutations in *ALS1*, while three had homozygous Pro‐197‐Thr mutations in *ALS2*. In the other population, five plants had homozygous Pro‐197‐Gln mutations in *ALS1*, two had homozygous Pro‐197‐Thr in *ALS2*, and one plant carried heterozygous Pro‐197‐Gln/Thr mutations, with Pro/Gln in *ALS1* and Pro/Thr in *ALS2*. The POAAN‐R4 population mostly carried Pro‐197‐Thr mutations, with one plant carrying a Trp‐574‐Leu substitution, both mutations were homozygous in *ALS1*. The POAAN‐R5 group (POAAN‐R5, POAAN‐R5.1 and POAAN‐R5.2) included plants with Trp‐574‐Leu substitutions in *ALS1* or *ALS2*, present as either homozygous or heterozygous mutations (Table 3). Lastly, all POAAN‐R6 plants carried homozygous Pro‐197‐Thr/Ala mutations, with the Ala substitution in *ALS1* and the Thr substitution in *ALS2*.

This study is the first to identify POAAN‐R3.1 with trans‐heterozygous Pro‐197‐Gln/Thr mutations (ratio of 2:1:1 (Pro/Gln/Thr)), POAAN‐R5.1 with heterozygous 3:1‐ratio Trp‐574‐Leu mutations, and POAAN‐R6 with trans‐heterozygous Pro‐197‐Thr/Ala mutations (ratio of 1:1 (Thr/Ala)) (Table 3). As expected, all plants tested from both R and S populations carried a fixed Ile‐1781‐Leu (ATA to A/T‐TA) substitution in the *ACCase* gene (data not shown).

### 3.2 Dose–response to ALS SU and SCT inhibitors

**Figure 1:**
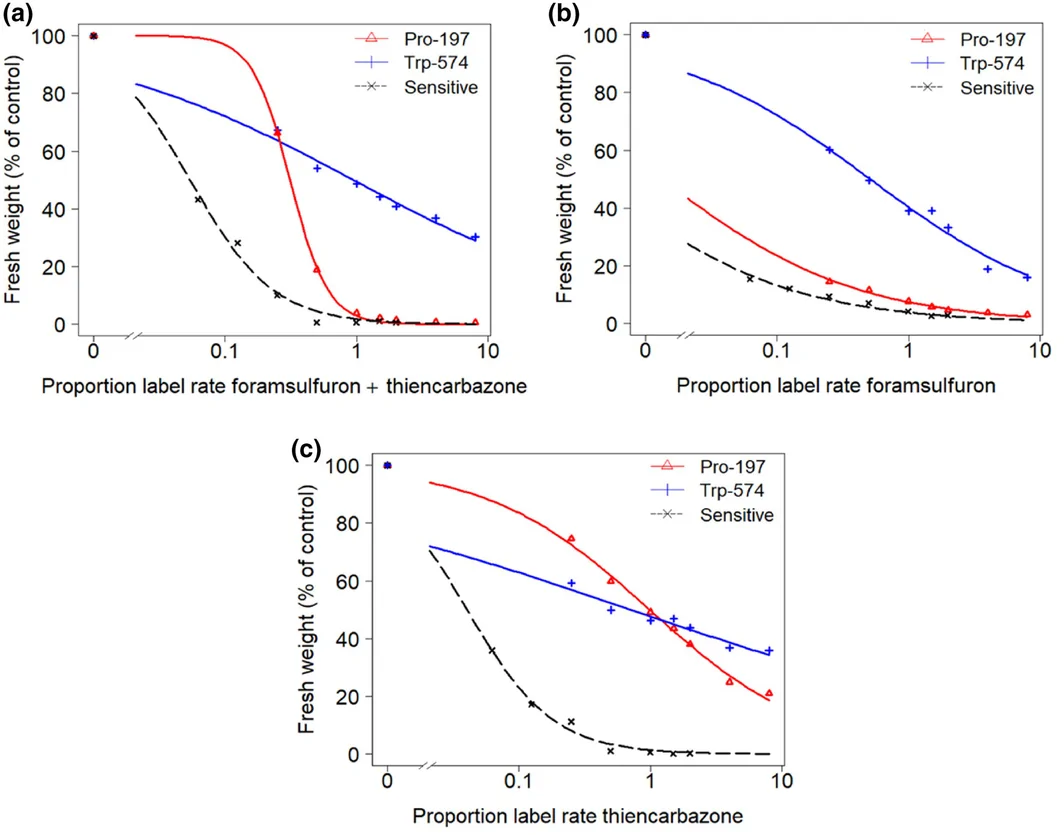
Dose–response curves of sensitive and resistant populations (grouped by mutation type: nine populations with Pro‐197 and three populations with Trp‐574) of *Poa annua* treated with a range of rates expressed as proportion of the recommended label rate of the co‐formulated foramsulfuron (SU) + thiencarbazone (SCT) (a) and the stand‐alone herbicides foramsulfuron (b) and theincarbazone (c).

Levels of response to stand‐alone herbicides foramsulfuron and thiencarbazone, as well as to the co‐formulation, were broadly determined by the ALS mutation present (Pro‐197 or Trp‐574) (Table 4). Dose–response analyses therefore compared pooled data from nine Pro‐197 populations (POAAN‐R1 to POAAN‐R4 and POAAN‐R6) and three Trp‐574 populations (POAAN‐R5, POAAN‐R5.1 and POAAN‐R5.2) with a S population (Fig. 1).

**Table 4:** Shoot fresh weight GR_50_ values (standard errors) of sensitive and resistant *Poa annua* populations harboring either Pro‐197 or Trp‐574 mutations treated with a range of rates of acetolactate synthase (ALS)‐inhibiting co‐formulated foramsulfuron (SU) + thiencarbazone (SCT) (50 + 30 g a.i. ha^−1^) and the stand‐alone herbicides foramsulfuron (45 g a.i. ha^−1^) and thiencarbazone (30 g a.i. ha^−1^)

		Co‐formulated				Stand‐alone							
		Foramsulfuron + thiencarbazone				Foramsulfuron				Thiencarbazone			
Mutation group	Population	GR_50_ (g a.i. ha^−1^)	RI	Mean GR_50_ (g a.i. ha^−1^)	Mean RI	GR_50_ (g a.i. ha^−1^)	RI	Mean GR_50_ (g a.i. ha^−1^)	Mean RI	GR_50_ (g a.i. ha^−1^)	RI	Mean GR_50_ (g a.i. ha^−1^)	Mean RI
Pro‐197	POAAN‐R1	15.0 (0.5) + 9.0 (0.4)	5.6	**15.6 (0.2) + 9.4 (0.2)**	**5.8**	0.6 (0.2)	3.0	**0.6 (0.1)**	**3.0**	29.9 (5.2)	24.9	**29.3 (1.6)**	**24.4**
	POAAN‐R1.1	16.4 (0.6) + 9.8 (0.4)	6.1			0.5 (0.1)	2.5			32.2 (6.4)	26.8		
	POAAN‐R1.2	15.3 (0.8) + 9.2 (0.5)	5.7			0.8 (0.2)	4.0			30.5 (4.2)	25.3		
	POAAN‐R1.3	16.1 (0.9) + 9.7 (0.6)	6.0			0.5 (0.1)	2.5			29.7 (5.0)	24.3		
	POAAN‐R2	16.5 (0.9) + 9.9 (0.5)	6.1			0.7 (0.2)	3.5			40.0 (6.3)	33.3			
	POAAN‐R3	14.8 (0.6) + 8.9 (0.3)	5.5			0.5 (0.1)	2.5			25.6 (3.5)	21.3		
	POAAN‐R3.1	14.8 (0.6) + 8.9 (0.4)	5.5			0.5 (0.1)	2.5			25.1 (4.2)	20.9		
	POAAN‐R4	15.0 (0.6) + 9.0 (0.4)	5.6			0.7 (0.2)	3.5			27.4 (4.2)	22.8		
	POAAN‐R6	16.6 (0.8) + 9.9 (0.5)	6.2			0.7 (0.2)	3.5			27.0 (4.2)	22.5		
Trp‐574	POAAN‐R5	39.0 (6.9) + 23.4 (4.2)	14.4	**46.7 (5.7) + 28.0 (3.4)**	**17.3**	20.7 (1.6)	103.5	**23.0 (1.2)**	**115.0**	17.4 (9.3)	14.5	**21.4 (5.1)**	**17.7**
	POAAN‐R5.1	55.7 (10.8) + 33.4 (6.5)	20.6			25.0 (1.9)	125			23.8 (6.7)	19.8		
	POAAN‐R5.2	48.9 (10.6) + 29.4 (6.3)	18.1			23.7 (1.8)	118.5			21.6 (11.2)	18.0		
Wild‐type	Sensitive	2.7 (0.4) + 1.7 (0.2)	—			0.2 (0.1)	—			1.2 (0.29)	‐		

*Note:* Resistance index (RI) was calculated as the ratio of GR_50_ values of resistant (R) and sensitive (S) and only one RI value is presented. Mean GR_50_ values (and RI) for populations grouped by mutation type are shown in bold. GR_50_, effective dose rate required to reduce growth by 50% relative to untreated plants; SU, sulfonylurea; SCT, sulfonylamino‐carbonyl‐triazolinone.

The S population was effectively controlled at 0.5 times the recommended label rate of both foramsulfuron + thiencarbazone and thiencarbazone alone, with biomass reductions exceeding 90% (Fig. 1). Corresponding GR_50_ values were 2.7 + 1.7 g ha^−1^ and 1.2 g ha^−1^, respectively (Table 4). The S population was even more sensitive to foramsulfuron alone, achieving over 90% biomass reduction at just 0.25 times the recommended label rate, with a GR_50_ of 0.2 g ha^−1^.

In contrast, the co‐formulated herbicide exhibited reduced efficacy on the Pro‐197 group (Fig. 1(a) and Table 4), with a few damaged but surviving plants at or above the recommended label rate (GR_50_ values ranging from 14.8 + 8.9 to 16.6 + 9.9 g ha^−1^; RI = 5.5–6.2). This reflects the contrasting responses to its active ingredients: high sensitivity to foramsulfuron alone (GR_50_ values of 0.5 to 0.8 g ha^−1^; RI = 2.5–4), effectively controlling plants at or below the recommended label rate (Fig. 1(b) and Table 4), and strong resistance to thiencarbazone alone (GR_50_ values of 25.1 to 40 g ha^−1^; RI = 20.9–33.3), with lack of control even at eight times the recommended label rate (Fig. 1(c) and Table 4). Mean GR_50_ values were 15.6 + 9.4 g ha^−1^ (RI = 5.8) for the co‐formulation, 0.6 g ha^−1^ (RI = 3) for foramsulfuron alone and 29.3 g ha^−1^ (RI = 24.4) for thiencarbazone alone.

The Trp‐574 group exhibited high cross‐resistance to all treatments (Fig. 1(a)–(c) and Table 4), with lack of control even at eight times the recommended label rate. GR_50_ values ranged from 39 + 23.4 to 55.7 + 33.4 g ha^−1^ (RI = 14.4–20.6) for the co‐formulation, 20.7–25 g ha^−1^ (RI = 103.5–125) for foramsulfuron, and 17.4–23.8 g ha^−1^ (RI = 14.5–19.8) for thiencarbazone. Mean GR_50_ values were 46.7 ± 28, 23 and 21.4 g ha^−1^, respectively (RI >17).

### 3.3 Herbicide interaction analysis

The ADM and Bliss Independence models were used to evaluate the efficacy of co‐formulated foramsulfuron + thiencarbazone relative to its individual active ingredients, on R and S populations (Table 5). Populations harbouring Trp‐574 mutations exhibited high resistance (RI >17) to all treatments (Table 4); thus, model results for this group were not interpreted further.

**Table 5:** Comparison of observed and expected biomass reduction (%) using dose–response analysis based on the Additive Dose Model (ADM) for sensitive and resistant *Poa annua* populations treated with foramsulfuron, thiencarbazone, and the co‐formulation

Population	Proportional label rate	Expected_additive^†^	Observed^‡^	Difference	*t*‐Statistic (*P* values)
Pro‐197 group	Untreated control	0	0	—	—
	0.25	89.07	33.89	−55.18	−21.28 (0.0002)
	0.5	93.42	81.29	−12.14	−5.21 (0.014)
	1.0	96.30	96.20	−0.10	−0.22 (0.84)
	1.5	97.42	97.88	0.46	0.52 (0.64)
	2.0	98.02	98.55	0.52	1.45 (0.24)
	4.0	98.97	99.14	0.17	0.88 (0.44)
	8.0	99.45	99.34	−0.11	−0.99 (0.40)
	Mean difference			−8.30	−1.21 (0.27)
Sensitive	Untreated control	0	0	‐	‐
	0.0625	94.51	56.76	−37.74	−7.03 (0.09)
	0.125	97.74	71.87	−25.87	−3.24 (0.19)
	0.25	99.23	90.06	−9.17	−5.61 (0.11)
	0.5	99.79	99.35	−0.44	−2.37 (0.25)
	1.0	99.97	99.35	−0.62	−3.32 (0.19)
	1.5	99.99	98.97	−1.02	−3.66 (0.17)
	2.0	100	99.44	−0.57	−6.08 (0.10)
	Mean difference			−9.49	−1.84 (0.11)

^†^ Predicted biomass reduction at each dose (*R*_Bliss_) derived from the Bliss Independence model within the ADM.

^‡^ Observed biomass reduction from the co‐formulation (*O*_mix_).

*Note:* The resistant group comprises nine populations (POAAN‐R1 to POAAN‐R5) carrying Pro‐197 mutations.

In the Pro‐197 group, no clear interaction pattern was observed (Table 5). Although the overall mean difference between observed (co‐formulated treatment) and expected biomass reduction suggested slight antagonism, this was not statistically significant (*P* > 0.05). Nevertheless, significant biomass reductions (*P* < 0.05) at 0.25× and 0.5× label rates corresponded with glasshouse phenotypic data, indicating relatively weaker efficacy of the co‐formulation (Table 5).

The S population showed a similar trend to the Pro‐197 group at lower dose rates (0.0625× to 0.25×) suggesting potential antagonism, though this was not statistically significant (*P* > 0.05) (Table 5). At dose rates equal to or above 0.5×, observed effects are likely due to random variation rather than true interactions between the herbicide components.

### 3.4 Structural modelling and ligand docking

**Figure 2:**
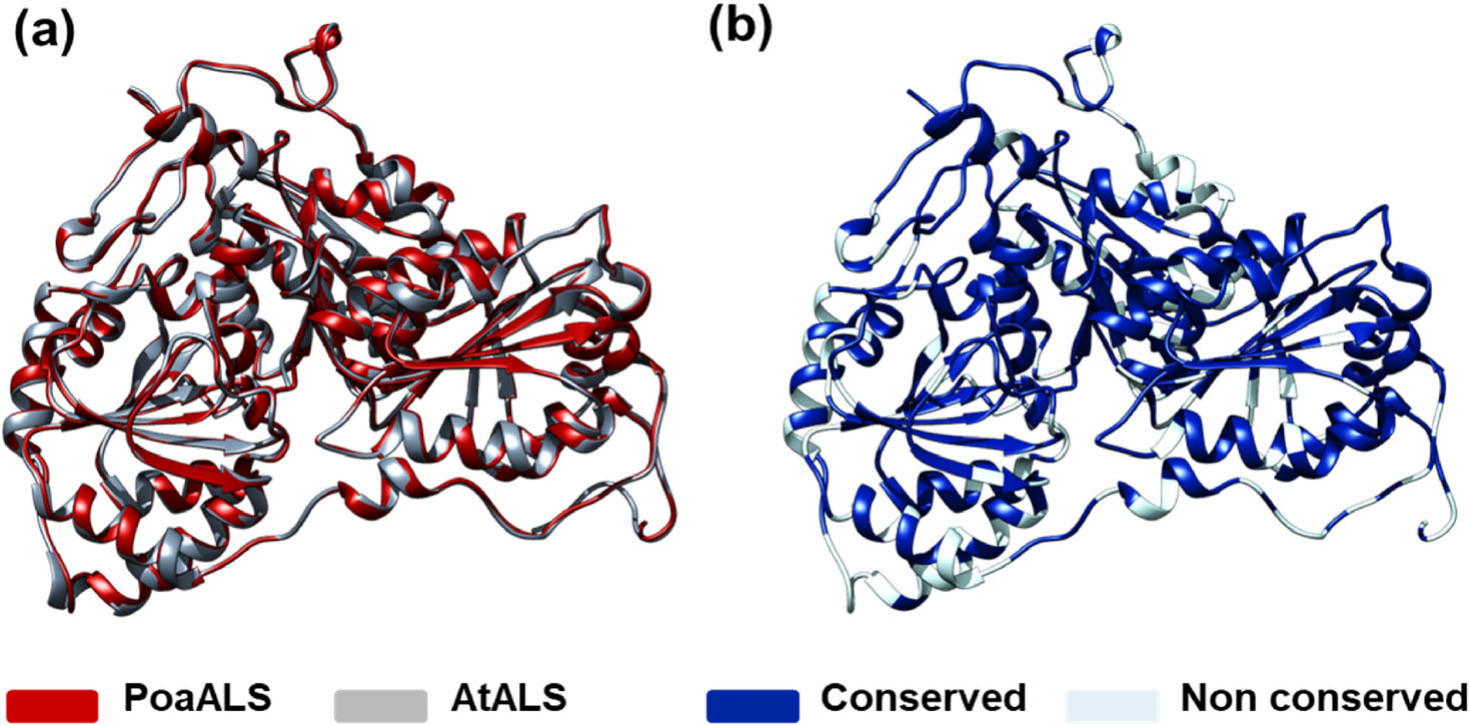
ALS from *Poa annua* and *Arabidopsis thaliana* share high sequence‐structure similiarity. The 3D superimposition of the modelled PoaALS structure (red) with the crystal structure of AtALS (grey; PDB ID: 3E9Y) (a). PoaALS model coloured according to residue conservation relative to the AtALS sequence (b).

The PoaALS amino acid sequence exhibits high homology with AtALS, especially in the catalytic domain and herbicide‐binding region. Using the AtALS crystal structure (PDB ID: 3E9Y) as a template, a PoaALS homology model was built for structural and ligand docking analyses (Fig. 2). Structural assessment of the resulting models revealed the high quality of the modelled structures (Fig. S4 and Table S1).

**Figure 3:**
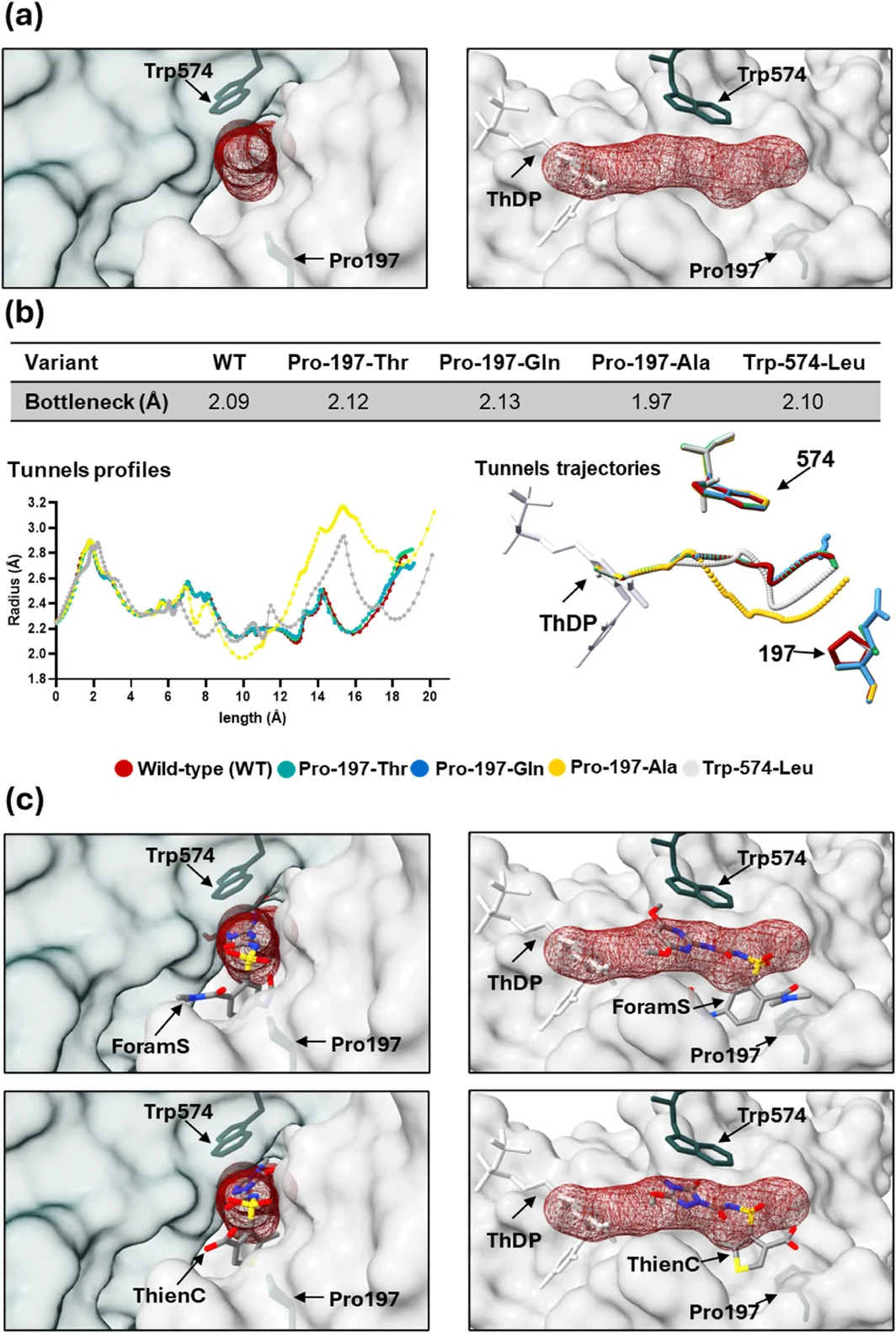
Identification and comparative analysis of PoaALS variants tunnels. (a) Surface representation of PoaALS‐wild type (WT) dimer (solid, grey) and the catalytic tunnel (mesh, red) in front (left) and side (right) views. Residues and ThDP are represented as sticks. (b) Tunnel profile representation (left) of PoaALS variants with their calculated minimum bottlenecks in the upper panel. Trajectories of PoaALS variants tunnels are represented as sticks in the right panel. (c) Superposition of the PoaALS‐foramsulfuron (upper panels) and PoaALS‐thiencarbazone (bottom panels) docked complexes with PoaALS‐WT dimer (solid, grey) with the catalytic tunnel (mesh, red).

Initially, tunnel shape was visually inspected among the different variants (Fig. S5). While most amino acid substitutions did not affect the tunnel entrance, the Pro‐197‐Gln substitution appeared to narrow the tunnel, potentially affecting herbicide entry (Fig. S5). Tunnel identification and analysis were then performed using MOLE 2.0 to characterise the conserved catalytic tunnel accommodating SU and SCT herbicides in PoaALS models (Fig. 3(a)).^32^, ^33^, ^34^ The tunnels were modelled using co‐crystallised ThDP as the starting point, and their profiles and trajectories are shown in Fig. 3(b) (left and right panels, respectively). As previously described by Pang *et al*.,^35^ the ALS access tunnel is funnel‐shaped, with Trp‐574 and Pro‐197 residues located in the wider region near the entrance (Fig. 3). Comparison of tunnels among variants showed no significant differences in their minimum bottleneck radius (Fig. 3(b), middle panel). Variants Pro‐197‐Ala and Trp‐574‐Leu showed notable differences in tunnel trajectories relative to the wild‐type protein, especially at the entrance region, with altered bottleneck positioning and increased radius near the entrance. These changes may influence herbicide binding kinetics (Fig. 3(b)).

**Figure 4:**
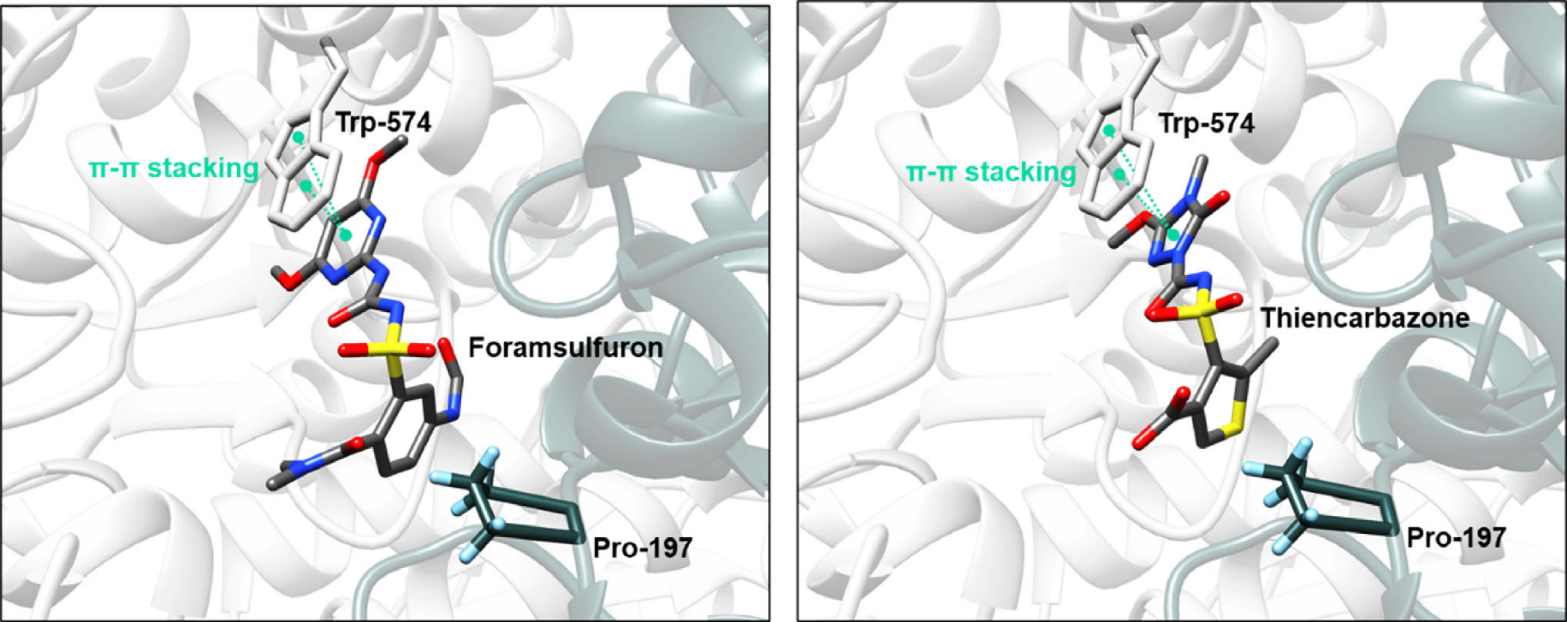
Docking of foramsulfuron and thiencarbazone in PoaALS‐wild type (WT). Foramsulfuron (left panel) and thiencarbazone (right panel) share the same location in PoaALSWT adopting a similar orientation according to the docking models. In both models, residues Trp‐574 and Pro‐197 are at a distance from the herbicides that allows the establishment of strong hydrophobic interactions, especially with the Trp‐574 whose aromatic ring can form π–π stacking interactions with both herbicides.

Docking analyses indicated that both foramsulfuron and thiencarbazone occupy the conserved ALS catalytic‐channel tunnel (Figs 3(c) and 4), consistent with previously reported SU and SCT binding modes.^32^, ^33^, ^34^ Despite not limiting the number of poses, six poses were obtained for foramsulfuron docking, with a > 1.6 difference in docking score (DS) between the minimum value pose (−5.94) and the next (−4.39) (Table 6). All poses formed π–π stacking interactions with Trp‐574, a key residue for ALS inhibitor binding.^32^, ^33^, ^34^ For thiencarbazone, ten poses were obtained, five with DS between −6.03 and − 5.29. The third pose (−5.43) showed an abrupt change in orientation and did not form π–π stacking with Trp‐574 and was discarded. The remaining four poses showed no major changes in orientation. MM/GBSA calculations were then used to refine the energetic ranking of the docked complexes and prioritise binding poses, reducing false positives inherent to docking scoring function (Table 6). For foramsulfuron, the selected pose had the lowest DS and the most favourable MM/GBSA energy, indicating agreement between the two ranking approaches (Table 6). Conversely, the lowest DS thiencarbazone pose (−6.03) showed an unfavourable MM/GBSA binding free energy (1.86 kcal mol^−1^) and was excluded. The second lowest DS pose (−5.80) showed the most favourable MM/GBSA binding energy and was selected (Table 6). Foramsulfuron‐docked complexes exhibited significantly lower free energies than thiencarbazone complexes, except for the selected thiencarbazone pose (ThienC 2) (Table 6). Overall, the MM/GBA analysis suggested that foramsulfuron should exhibit a stronger binding affinity than thiencarbazone, with binding free energies of −33.37 kcal mol^−1^ and −22.58 kcal mol^−1^, respectively.

**Table 6:** Comparison of docking score and Prime Molecular Mechanics/Generalised Born Surface Area (MM/GBSA) binding energy values for the acetolactate synthase (ALS)‐herbicide docked poses

Pose	Docking score	π–π Stacking Trp574	MM/GBSA ΔGbind (kcal mol^−1^)	MM/GBSA Δcoul (kcal mol^−1^)	MM/GBSA Δcov (kcal mol^−1^)	MM/GBSA ΔH‐bond (kcal mol^−1^)	MM/GBSA Δlipo (kcal mol^−1^)	MM/GBSA ΔvdW (kcal mol^−1^)
ForamS 1	−5.937	Yes	−33.37	40.71	2.81	−4.66	−13.33	−36.69
ForamS 2	−4.385	Yes	−21.22	50.20	12.75	−4.45	−9.11	−45.63
ForamS 3	−4.007	Yes	−14.65	57.75	13.55	−3.84	−8.46	−40.98
ForamS 4	−3.282	Yes	−25.76	54.73	7.29	−4.02	−8.50	−43.60
ForamS 5	−3.161	Yes	−31.14	47.72	7.67	−3.67	−8.26	−50.10
ForamS 6	−2.835	Yes	−26.71	42.77	0.87	−3.91	−12.96	−30.11
ThienC 1	−6.033	Yes	1.86	189.48	13.07	−2.79	−10.16	−40.27
ThienC 2	−5.800	Yes	−22.58	174.93	1.09	−3.06	−10.01	−40.79
ThienC 3	−5.430	No	8.79	184.51	2.89	−3.60	−5.09	−25.65
ThienC 4	−5.390	Yes	−10.26	199.59	−0.87	−2.23	−12.03	−44.21
ThienC 5	−5.292	Yes	−8.28	211.32	2.76	−1.51	−10.27	−53.35
ThienC 6	−4.898	No	−8.08	182.16	1.95	−2.88	−8.13	−40.29
ThienC 7	−4.775	Yes	4.65	196.56	2.71	−2.14	−10.27	−30.03
ThienC 8	−4.427	No	−15.28	158.47	3.09	−4.28	−6.75	−31.40
ThienC 9	−3.859	No	−16.54	188.49	2.51	−2.41	−5.22	−38.43
ThienC 10	−2.807	No	−16.02	195.56	−0.45	−2.47	−6.95	−40.98

Abbreviations: binding free energy (Gbind); Coulomb energy (coul); covalent binding energy (cov); hydrogen‐bonding correction (H‐bond); lipophilic energy (lipo); van der Waals energy (vdW); foramsulfuron (ForamS); thiencarbazone (ThienC).

**Figure 5:**
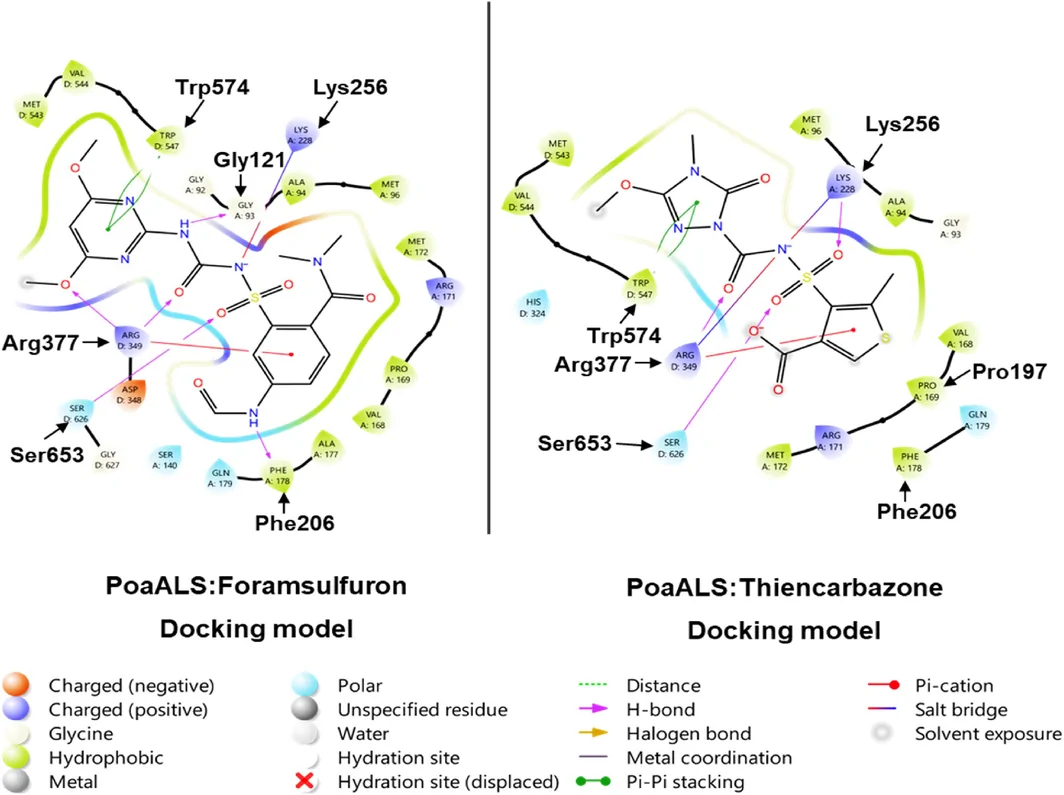
Ligand interaction diagram of herbicides–ALS docked complexes. A higher number of interactions were established between PoaALS and foramsulfuron than with thiencarbazone, including strong interactions involving residues Trp‐574, Arg‐377 and Lys‐256 for both herbicides. Residue numbering of ligand interaction diagrams correspond to the PoaALS sequence. The equivalent residues in the AtALS sequence are indicated with black arrows.

In the selected final poses, both herbicides adopt extended conformations along the tunnel, stabilised by networks of polar and non‐polar interactions (Figs 3 and 5). The N‐containing ring of the herbicides inserts into a hydrophobic pocket, establishing π–π stacking with Trp‐574, a key residue for ALS inhibitor binding.^32^, ^33^ This explains why Trp‐574‐Leu variants are resistant to both herbicides, as the stabilising π–π interaction crucial for herbicide binding cannot be established with leucine, in contrast to tryptophan (Figs 4 and 5). Additional stabilising interactions include π–cation contacts with Arg‐377 and electrostatic and hydrogen‐bond interactions involving Arg‐377, Lys‐256, and Ser‐653 (Fig. 5). Foramsulfuron forms a more extensive hydrogen‐bond and electrostatic network than thiencarbazone, involving Gly‐121, Phe‐206, Arg‐377, Ser‐653, Trp‐574, and Lys‐256, mediated by the SU bridge and associated urea functionalities. Thiencarbazone exhibits a slightly reduced interaction network, involving the same residues as foramsulfuron, except for Gly‐121 and Phe‐206. This may be, at least in part, due to the slight but significant difference in the free energy values calculated by MM/GBSA for the selected docked complexes of foramsulfuron (−33.37 kcal mol^−1^) and thiencarbazone (−22.58 kcal mol^−1^).

## 4 DISCUSSION

Herbicide co‐formulations are widely used in modern weed management to enhance efficacy and broaden control spectra in response to evolving resistance.^36^ However, such mixtures are often assumed to delay resistance evolution without rigorous comparison to equally effective stand‐alone doses or systematic assessment of mixture interactions.^37^, ^38^ While trade‐offs are generally negligible when targeting sensitive weeds, they can become critical in target‐site R populations, where interaction effects may reduce performance, increase survivors and accelerate resistance spread.^31^

Our study confirms these concerns: the SU + SCT co‐formulation foramsulfuron + thiencarbazone provides limited control of *Poa annua* carrying Pro‐197 ALS mutations, especially at or below the recommended label rate. Consequently, the efficacy of the co‐formulation is likely reduced in fields where ALS‐resistant *Poa annua* populations are present. These findings are consistent with previous reports showing that this co‐formulation did not provide complete control of ALS‐resistant weeds, including *Alopecurus myosuroides*, *E. crus‐galli*, *Papaver rhoeas* L. (common poppy), *Stellaria media* L. (common chickweed) or *Matricaria inodora* L. (scentless mayweed).^39^, ^40^

In *Poa annua* and other tetraploid species, where *ALS1* and *ALS2* gene variants are expressed at similar levels, mutations in either homologue do not differentially affect resistance levels or cross‐resistance patterns.^23^, ^41^, ^42^ Consistent with this, all Pro‐197 substitutions examined here (Thr, Gln or Ala) exhibited a common response profile: reduced efficacy of the SU + SCT co‐formulation, retained sensitivity to foramsulfuron and high resistance to thiencarbazone. The foramsulfuron susceptibility observed in R and S populations of Pro‐197‐resistant *Poa annua* in this study, as well as in *L. multiflorum* in a separate study (Vijayakumar *et al*. unpublished; Fig. S1), was more pronounced than that reported in a Pro‐197 mutant *Poa annua* population from non‐arable systems.^20^ Interaction analysis indicated an antagonistic trend at rates below the recommended label rate (*P* < 0.05), but this effect was not significant overall, suggesting that foramsulfuron plays the dominant role in co‐formulation performance, partially compensating for the poor partner activity of thiencarbazone. Interestingly, this consistent response contrasts with our previous work in *Poa annua*, where differential resistance patterns to SU and TP chemistries were observed depending on the specific Pro‐197 substitution (Gln or Thr).^5^ As expected, Trp‐574 populations conferred broad ALS cross‐resistance.^20^, ^22^

ALS is a multimeric enzyme in which substrate access to the catalytic site occurs via a conserved channel, where herbicides bind within a site formed by adjacent subunits at the interface between protomers.^34^ Pro‐197 is located at the end of an α‐helix near the entrance of the access tunnel, whereas Trp‐574 lies closer to the active site and plays a key role in shaping the channel and influencing herbicide interactions.^33^ Structural modelling of PoaALS allowed us to study the channel geometries among Pro‐197 variants. While initial visual inspections suggested a narrower entrance tunnel in Pro‐197‐Gln variant, careful quantitative analysis by MOLE 2.0, allowed this to be ruled out and identified Pro‐197‐Ala and Trp‐574‐Leu as the only variants showing a significant difference in tunnel geometry compared to the wild‐type PoaALS. Taking into account that the geometry of the herbicide entrance tunnel in most Pro‐197 variants is only slightly affected, a potential effect of these mutations on herbicide entry is questionable. Only in the case of the Pro‐197‐Ala substitution was a wider channel entrance detected, which might have consequences for herbicide entry and residence time within the tunnel. Similar effects have also been reported in *Lolium perenne* L. (perennial ryegrass), where Pro‐197 substitutions at the channel entrance reduce binding affinity via disruption of hydrogen and hydrophobic interactions and local structural perturbations, resulting in weaker enzyme–herbicide interactions.^43^

Further docking analysis showed that foramsulfuron and thiencarbazone occupy overlapping tunnel regions with similar binding poses and key stabilising interactions, including π–π stacking with Trp‐574 in PoaALS. Foramsulfuron forms slightly more polar interactions than thiencarbazone, including hydrogen bonds with Phe‐206 and Gly‐121, and MM/GBSA calculations suggest a higher affinity for foramsulfuron than thiencarbazone for PoaALS, consistent with Garcia *et al*.^34^ However, how these predicted quantitative differences might explain the pronounced thiencarbazone resistance observed in Pro‐197 variants remains unclear. Interestingly, Lonhienne *et al*.^44^ indicate that ALS herbicides act at two levels: (a) blocking substrate access to the catalytic pocket by obstructing the entrance tunnel, and (b) inducing accumulative enzyme inhibition via oxidative inactivation. It has been demonstrated that Pro‐197 variants have a differential effect on this accumulative inhibition across ALS herbicide families.^44^ How this mechanism is affected by foramsulfuron and thiencarbazone in PoaALS Pro‐197 variants is still unknown but may provide a useful starting point for explaining the different activities of these two ALS herbicide families in Pro‐197 *Poa annua* populations.

POAAN‐R2 is the only population harbouring the Pro‐197‐Gln mutation and exhibits the highest level of resistance (40 g ha^−1^) to thiencarbazone compared with other Pro‐197 mutant populations (25.1–32.2 g ha^−1^). This suggests that Pro‐197‐Gln might selectively impair thiencarbazone binding, while foramsulfuron remains largely unaffected, as evidenced by the similar sensitivity to foramsulfuron across Pro‐197 mutant populations. Accordingly, while docking captures conserved binding frameworks and hydrogen‐bonding differences, it does not fully reflect mutation‐dependent steric or dynamic effects within the access channel. The more extensive interaction network of foramsulfuron, supported by free energy calculations based on MM/GBSA, likely allows it to tolerate subtle geometric constraints, whereas thiencarbazone appears more sensitive. This mechanistic explanation is consistent with dose–response data, where the GR_50_ of foramsulfuron (0.2 g ha^−1^) was six‐fold lower than that of thiencarbazone (1.2 g ha^−1^), reflecting intrinsically stronger binding and inhibition of the wild‐type ALS enzyme. In contrast, Trp‐574‐Leu located deeper in the tunnel, disrupts critical π–π stacking accounting for broad ALS cross‐resistance.^32^, ^33^

Foramsulfuron efficacy may be analogous to the ACCase inhibitor clethodim. Both herbicides are relatively difficult to metabolise, retain activity on R populations carrying specific TSR (ALS Pro‐197 or ACCase Ile‐1781) mutations and exhibit resistance primarily driven by mutation‐specific effects (ALS Trp‐574 or ACCase Asp‐2078), with some exceptions.^20^, ^45^, ^46^ Our docking and structural analyses suggest that foramsulfuron efficiently accommodates the ALS access tunnel in Pro‐197 variants, explaining its retained activity in *Poa annua* and potentially in other grass weeds carrying the same mutations, such as *L. multiflorum*, where resistance to foramsulfuron + thiencarbazone, but not to foramsulfuron, was noted (Vijayakumar *et al*. unpublished; Fig. S1). In contrast, foramsulfuron efficacy, whether applied alone or in co‐formulation, is limited in broadleaf species such as *Papaver rhoeas* carrying a Pro‐197 mutation,^47^ highlighting a species‐specific component of Pro‐197‐mediated resistance.

From a practical perspective, the efficacy of the SU + SCT co‐formulation, developed for ALS‐tolerant beet, on grass weeds, is likely compromised in fields with species carrying Pro‐197 mutations. These observations highlight the importance of considering access‐channel geometry and ligand interaction networks, beyond static docking, when evaluating herbicide performance on target‐site resistant weeds. By integrating genetic characterisation, dose–response analysis, and computational modelling, this study provides initial mechanistic insights into the differential response of foramsulfuron and thiencarbazone against ALS‐resistant *Poa annua*.

## CONFLICT OF INTEREST

The authors declare no conflicts of interest.

## Supporting information


**Figure S1.** Symptoms of the sensitive and Pro‐197‐resistant (LOLMU‐R) *Lolium multiflorum* populations following application of four ALS‐inhibiting herbicides: mesosulfuron + iodosulfuron, pyroxsulam, foramsulfuron + thiencarbazone, and foramsulfuron. LOLMU‐R was treated at dose rates ranging from 0.25× to 8×, while the sensitive population was treated at 0.0625× to 2× the recommended label rate (highlighted in yellow). This work forms part of a manuscript currently being finalised for submission (Vijayakumar *et al*. unpublished).
**Figure S2**. Symptoms of the sensitive and five resistant *Poa annua* populations (POAAN‐R1 to POAAN‐R5) following application of the ALS‐inhibiting herbicides mesosulfuron + thiencarbazone and stand‐alone thiencarbazone. Resistant populations were treated at dose rates ranging from 0.25× to 8×, while the sensitive population was treated at 0.0625× to 2× the recommended label rate (where 1× = 14.9 + 5 g ha^−1^ for the co‐formulation and 7.5 g ha^−1^ for thiencarbazone applied alone, corresponding to the maximum permitted rate in cereal crops; highlighted in yellow). Dose–response analysis of these representative populations to mesosulfuron + thiencarbazone has been reported previously.^5^
**Figure S3**. Symptoms of the sensitive and ten resistant *Poa annua* populations (POAAN‐R1 to POAAN‐R6; with sufficient seed quantity) following application of the ALS‐inhibiting herbicide mesosulfuron + iodosulfuron. Resistant populations were treated at dose rates ranging from 0.25× to 8×, while the sensitive population was treated at 0.0625× to 2× the recommended label rate (where 1× = 15 + 5 g ha^−1^; highlighted in yellow). Dose–response analysis of representative populations (POAAN‐R1, POAAN‐R2, POAAN‐R3, POAAN‐R4 and POAAN‐R5) has been reported previously.^5^
**Figure S4**. Ramachandran plots of PoaALS structural model variants.
**Table S1**. Validation metrics of PoaALS structural model variants.
**Figure S5**. Surface representation of the catalytic tunnel of PoaALS variants. Visual inspection of the Pro‐197‐Gln variant suggests a narrower tunnel entrance compared to the wild‐type (WT) protein. Both protomers contribute to tunnel formation and are shown in different intensities of the same colour.

## Data Availability

The data that support the findings of this study are available from the corresponding author upon reasonable request.
