# Supplementary material for: Pro‐197 mutations confer resistance to thiencarbazone but not to foramsulfuron in ALS‐resistant Poa annua populations
Source: Pest Manag Sci. 2026 Jun 21;82(10):10134–46. doi: 10.1002/ps.71051 (PMC13569366; doi:10.1002/ps.71051)
Supplement: Supplementary file 1 [file PS-82-10134-s001.docx]

**Supplementary Information, Figure S1.** Symptoms of the sensitive and Pro-197-resistant (LOLMU-R) *L. multiflorum* populations following application of four ALS-inhibiting herbicides: mesosulfuron + iodosulfuron, pyroxsulam, foramsulfuron + thiencarbazone, and foramsulfuron. LOLMU-R was treated at dose rates ranging from 0.25x to 8x, while the sensitive population was treated at 0.0625x to 2x the recommended label rate (highlighted in yellow). This work forms part of a manuscript currently being finalised for submission (Vijayakumar *et al.* unpublished).

**Supplementary Information, Figure S2.** Symptoms of the sensitive and five resistant *P. annua* populations (POAAN-R1 to POAAN-R5) following application of the ALS-inhibiting herbicides mesosulfuron + thiencarbazone and stand-alone thiencarbazone. Resistant populations were treated at dose rates ranging from 0.25x to 8x, while the sensitive population was treated at 0.0625x to 2x the recommended label rate (where 1x = 14.9 + 5 g ha^-1^ for the co-formulation and 7.5 g ha^-1^ for thiencarbazone applied alone, corresponding to the maximum permitted rate in cereal crops; highlighted in yellow). Dose-response analysis of these representative populations to mesosulfuron + thiencarbazone has been reported previously.^5^

**Supplementary Information, Figure S3.** Symptoms of the sensitive and ten resistant *P. annua* populations (POAAN-R1 to POAAN-R6; with sufficient seed quantity) following application of the ALS-inhibiting herbicide mesosulfuron + iodosulfuron. Resistant populations were treated at dose rates ranging from 0.25x to 8x, while the sensitive population was treated at 0.0625x to 2x the recommended label rate (where 1x = 15 + 5 g ha^-1^; highlighted in yellow). Dose-response analysis of representative populations (POAAN-R1, POAAN-R2, POAAN-R3, POAAN-R4 and POAAN-R5) has been reported previously.^5^

**Supplementary Information, Figure S4.** Ramachandran plots of PoaALS structural model variants

**Supplementary Information, Table S1.** Validation metrics of PoaALS structural model variants

| Model | Sequence identity | QMEANDisCo score | Molprobity score | Clash score |
| --- | --- | --- | --- | --- |
| Wild-type | 75.65 % | 0.87 ± 0.05 | 1.73 | 2.63 |
| Pro-197-Thr | 75.47 % | 0.87 ± 0.05 | 1.67 | 1.94 |
| Pro-197-Gln | 75.47 % | 0.87 ± 0.05 | 1.68 | 1.94 |
| Pro-197-Ala | 74.96 % | 0.86 ± 0.05 | 1.59 | 1.88 |
| Trp-574-Leu | 75.61 % | 0.86 ± 0.05 | 1.64 | 1.94 |

**Supplementary Information, Figure S5.** Surface representation of the catalytic tunnel of PoaALS variants. Visual inspection of the Pro-197-Gln variant suggests a narrower tunnel entrance compared to the wild-type (WT) protein. Both protomers contribute to tunnel formation and are shown in different intensities of the same colour.
